# Recurrence prediction in clear cell renal cell carcinoma using machine learning of quantitative nuclear features

**DOI:** 10.1038/s41598-023-38097-7

**Published:** 2023-07-07

**Authors:** Shuya Matsubara, Akira Saito, Naoto Tokuyama, Ryu Muraoka, Takeshi Hashimoto, Naoya Satake, Toshitaka Nagao, Masahiko Kuroda, Yoshio Ohno

**Affiliations:** 1 Department of Urology, Tokyo Medical University Hospital, 6-7-1, Nishi-Shinjuku, Shinjuku-Ku, Tokyo, 160-0023 Japan; 2 Department of AI Applied Quantitative Clinical Science, Tokyo Medical University, 6-1-1, Shinjuku, Shinjuku-Ku, Tokyo, 160-8402 Japan; 3 Department of Molecular Pathology, Tokyo Medical University, 6-1-1, Shinjuku, Shinjuku-Ku, Tokyo, 160-8402 Japan; 4 Department of Anatomic Pathology, Tokyo Medical University, 6-1-1, Nishi-Shinjuku, Shinjuku-Ku, Tokyo, 160-8402 Japan

**Keywords:** Renal cell carcinoma, Computational models, Oncology

## Abstract

The recurrence of non-metastatic renal cell carcinoma (RCC) may occur early or late after surgery. This study aimed to develop a recurrence prediction machine learning model based on quantitative nuclear morphologic features of clear cell RCC (ccRCC). We investigated 131 ccRCC patients who underwent nephrectomy (T1-3N0M0). Forty had recurrence within 5 years and 22 between 5 and 10 years; thirty-seven were recurrence-free during 5–10 years and 32 were for more than 10 years. We extracted nuclear features from regions of interest (ROIs) using a digital pathology technique and used them to train 5- and 10-year Support Vector Machine models for recurrence prediction. The models predicted recurrence at 5/10 years after surgery with accuracies of 86.4%/74.1% for each ROI and 100%/100% for each case, respectively. By combining the two models, the accuracy of the recurrence prediction within 5 years was 100%. However, recurrence between 5 and 10 years was correctly predicted for only 5 of the 12 test cases. The machine learning models showed good accuracy for recurrence prediction within 5 years after surgery and may be useful for the design of follow-up protocols and patient selection for adjuvant therapy.

## Introduction

Renal cell carcinoma (RCC) is the most common malignant tumor of the kidney. Partial or radical nephrectomy is the standard treatment for localized RCC. However, it was reported that 20–30% of patients with localized disease had recurrence after nephrectomy^1^. Approximately three-quarter of patients, who had developed recurrence, were identified during the first 5 years after surgery. The remaining quarter of patients developed recurrence more than 5 years after nephrectomy^2^.

The American Urologic Association (AUA), National Comprehensive Cancer Network (NCCN), and European Association of Urology (EAU) have each published guidelines to recommend follow-up protocol based on risk classification^3–5^. The AUA guideline recommends classifying the patients who had undergone nephrectomy into 4 risk groups based on pathological Tumor stage (pT) and nuclear grade (low, intermediate, high, and very high-risk group). The patients should be checked up under follow-up protocol recommended in each risk group. The NCCN Guideline version 2.2023 recommends a similar follow-up protocol based on TNM stage. The EAU guideline recommends risk-based follow-up protocol using the 2003 Leibovich model for patients with clear cell RCC (ccRCC) or the University of California Los Angeles integrated staging system for the patients with non-ccRCC^6, 7^.

However, definitive follow-up protocol beyond 5 years is not described in all three guidelines. The clinician may follow up the patients using abdominal scan every 6 months by patients and/or clinician’s preference even after the initial 5-year recurrence-free period. In addition, the NCCN guideline recommends adjuvant immunotherapy for patients with stage 2 and 3 diseases. Therefore, it is important to predict precise risk of recurrence considering time to recurrence.

In this study, we focused on quantitative nuclear morphologic features obtained digital pathological technique and developed novel prediction models using machine learning to determine risk of recurrence after surgery. Especially, for predicting risk of recurrence more than 5 years after surgery, we combined two recurrence prediction model at 5-year and 10-year after surgery.

## Results

### Patients’ characteristics and quantitative nuclear features

Patients’ characteristics are shown in Table 1. Regarding the time of recurrence, 40 patients had recurrence within 5 years (Group A) and 22 patients had recurrence between 5 and 10 years (Group B). Thirty-seven patients were recurrence-free during 5–10 years follow-up (Group C) and 32 patients were recurrence-free more than 10 years after surgery (Group D). There was significant difference in presentation mode, TNM stage, nuclear grade, and microscopic venous invasion among the 4 groups.

**Table 1:** Patients’ characteristics.

		All patients (n = 131)	Group				p value
			A (n = 40)	B (n = 22)	C (n = 37)	D (n = 32)	
Age	Median (range)	60 (51–65)	65 (58–69)	57 (43–61)	58 (51–68)	59 (50–63)	0.176
Gender	Male	92	27	15	24	26	0.918
	Female	39	13	7	13	6	
Presentation mode	Incidental	94	25	13	32	24	0.042
	Symptomatic	37	15	9	5	8	
Nephrectomy	Radical	107	36	22	20	29	< 0.001
	Partial	24	4	0	17	3	
TNM stage	1	95	17	13	35	30	< 0.001
	2	13	6	5	1	1	
	3	23	17	4	1	1	
Nuclear grade	1	42	10	7	14	11	0.044
	2	83	25	15	23	20	
	3 + 4	6	5	0	0	1	
Microscopic venous invasion	Negative	111	29	17	37	28	0.0004
	Positive	20	11	5	0	4	
ECOG-PS	0	127	39	19	37	32	0.072
	> = 1	4	1	3	0	0	
Follow-up period	Median (range)	Median	59 (38–82)	111 (96–136)	103 (93–107)	144 (138–153)	
Recurrence	Yes	62	40	22	0	0	
	No	69	0	0	37	32	

Group A: Recurrence within 5 years.

Group B: Recurrence between 5 and 10 years.

Group C: Recurrence-free with 5–10 years follow-up.

Group D: Recurrence-free for more than 10 years follow-up.

We obtained 4312 regions of interest (ROIs) from a total of 131 patients. We extracted 2,512,771 cell nuclei from total ROI. From each nucleus, 80 quantitative features were extracted, which were classified into nuclear shape related features and texture related features (Supplementary Table 1). The 80 nucleus features of each nucleus were converted into 960 features per ROI, such as mean, standard deviation, and heterogeneity by cell feature level co-occurrence matrix (CFLCM), and these ROI-based features were used for support vector machine (SVM) analysis.

### Development of recurrence prediction model using machine learning algorithm and validation

#### 5-year prediction model

A total of 131 patients were divided into 100 training and 31 test cases (Supplementary Table 2). SVM training was performed to optimize the prognostic accuracy. In training sets, classification of ROIs with regards to recurrence within 5-years indicated an accuracy of 92.7%. This model was validated using test sets; the accuracy of ROI classification was 86.4%. Aggregating the results of ROIs to the cases, the accuracy was 100% (Table 2). Supplementary Table 3 shows a summary of the top 20 features highly contributing to non-recurrence and recurrence in 5-year model.

**Table 2:** Training and test set results for the 5-year recurrence prediction model.

	Prediction		
	Rec ( +)	Rec ( −)	Total
(a) Training set result			
Accuracy: 92.7%			
Truth			
Rec ( +)	708	166	874
Rec ( −)	77	2378	2455
Total	785	2544	3329
(b) Test set result (ROI-based)			
Accuracy: 86.4%			
Truth			
Rec ( +)	222	53	275
Rec ( −)	81	627	708
Total	303	680	983
(c) Test set result (case-based)			
Accuracy: 100%			
Truth			
Rec ( +)	10	0	10
Rec ( −)	0	21	21
Total	10	21	31

#### 10-year prediction model

A total of 94 patients (Group A, B, and D) were randomly divided into 72 training and 22 test sets (Supplementary Table 4). In training set, the model was generated with an accuracy of 96.7% (Table 3). In the validation using test set, the accuracy for the ROIs was 74.1%. Aggregating the results of ROIs to the cases, the accuracy was 100%. Supplementary Table 5 shows a summary of the top 20 features highly contributing to non-recurrence and recurrence in 10-year model.

**Table 3:** Training and test set results for the 10-year recurrence prediction model.

	Prediction		
	Rec ( +)	Rec ( −)	Total
(a) Training set result			
Accuracy: 96.7%			
Truth			
Rec ( +)	1420	34	1454
Rec ( −)	44	845	889
Total	1464	879	2343
(b) Test set result (ROI-based)			
Accuracy: 74.1%			
Truth			
Rec ( +)	365	231	596
Rec ( −)	24	363	387
Total	389	594	983
(c) Test set result (case-based)			
Accuracy: 100%			
Truth			
Rec ( +)	14	0	14
Rec ( −)	0	8	8
Total	14	8	22

### Prediction of time of recurrence during postoperative course by combining 5-year and 10-year prediction models

**Figure 1:**
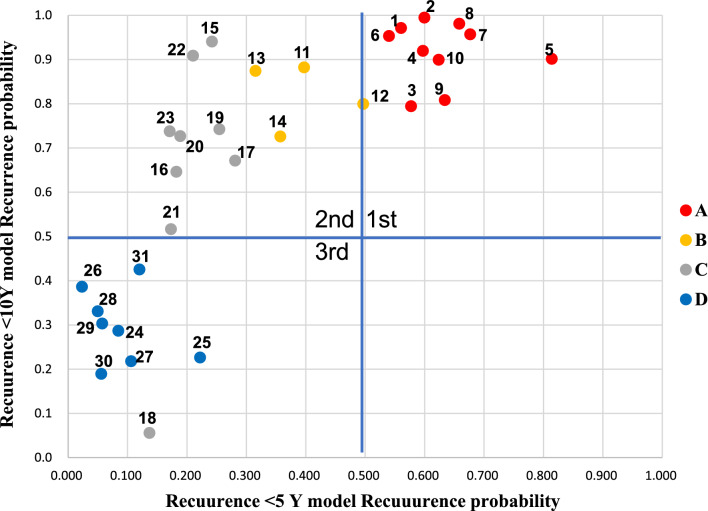
Probability of recurrence based on 5- and 10-year models in test cases. The vertical axis shows the probability of the 10-year recurrence prediction model and the horizontal axis shows the probability of the 5-year recurrence prediction model. For each model, probability > 0.50 is the cutoff for recurrence. First quadrant area shows recurrence risk of more than 50% by both prediction models; the patients are predicted to recur within 5 years. Second quadrant area shows recurrence risk of 50% or less by 5-year model and of more than 50% by 10-year model; the patients are predicted to recur between 5 and 10 years after surgery. Third quadrant area shows recurrence risk of 50% or less by both prediction models; the patients were predicted to be recurrence-free within 10 years after surgery. The color of the dots indicates status and time of recurrence in each test case. Group A (Red): the patients had recurrence within 5 years, Group B (yellow): the patients had recurrence between 5 and 10 years after surgery, Group C (gray): the patients had been recurrence-free during 5–10 years after surgery, and Group D (blue): the patients were recurrence-free more than 10 years after surgery.

We combined two models to predict time of recurrence during postoperative course. Figure 1 shows the recurrence probability calculated by 5- and 10-year models in each test case, which were used in the validation of 5-year model. All group A patients (red) who had recurrence within 5-years were plotted in the 1st quadrant area. All group B patients (yellow) who had recurrence between 5 and 10 years were plotted in the 2nd quadrant area. As for group C patients (grey) who were recurrence-free during 5–10 years, eight were plotted in the 2nd quadrant area and one was plotted in the 3rd quadrant area. All group D patients (blue) who had been recurrence-free for more than 10 years after surgery were plotted in the third quadrant area. None was plotted in the 4th quadrant area, which indicated the patients who had contradictory prediction in 5-year and 10-year models. The accuracy for prediction in group A, B, and D patients was 100%. However, the accuracy of prediction in group C patients could not be determined because the follow-up period was less than 10 years. Therefore, we tracked the status of recurrence to December 2021. In 3 patients, the follow-up ended before 10 years after surgery (at 104 months in case 15, at 113 months in case 16, and at 112 months in case 23). Six patients (case 17, 18, 19, 20, 21, 22) had been followed up for more than 120 months (range, 131–207 months). Case 22 in the 2nd quadrant area developed recurrence at 66 months after surgery and case 18 in 3rd quadrant area has been recurrence-free for more than 10 years. These 2 cases were correctly predicted. On the contrary, 4 cases (17, 19, 20, 21) did not develop recurrence between 5 and 10 years after surgery; false prediction was noted for these cases.

**Figure 2:**
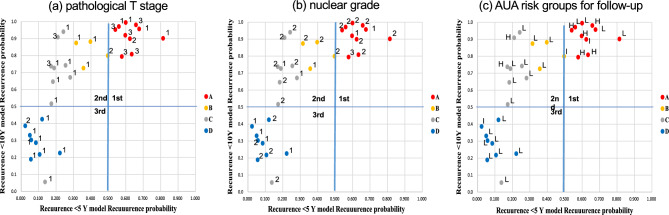
Association among T stage, nuclear grade, and probability of recurrence by 5- and 10-year prediction models. (**a**) T stage and probability of recurrence by 5- and 10-year prediction models. T1, 1; T2, 2; T3, 3. (**b**) Nuclear grade and probability of recurrence by 5- and 10-year prediction models. G1, 1; G2, 2; G3, 3. (**c**) The American Urologic Association (AUA) risk group for follow-up and probability of recurrence by 5- and 10-year prediction models.

Figure 2 shows the association among T stage, nuclear grade, AUA risk group for follow-up, and recurrence probability by 5- and 10-year prediction models. T stage, nuclear grade, AUA risk group for follow-up in each test case were distributed widely beyond the three area. No trend was noticed in the distribution of each factor. The Cox multivariate analyses demonstrated probability calculations for 5-year and 10-year recurrence prediction models were independent predictors for recurrence (Supplementary Table 6).

## Discussion

The development of digital pathology and artificial intelligence has been applied to various issues regarding pathological diagnosis and prognosis prediction. We previously reported the development and future potential of recurrence prediction models using machine learning of quantitative nuclear morphologic features in hepatocellular carcinoma and bladder cancer^8, 9^.

In this study, we developed a novel system to predict recurrence in patients with ccRCC using quantitative nuclear morphological features. Since ccRCC have few structural features, we focused on differences in nuclear morphology and chromatin texture. Using 90 quantitative nuclear morphologic features, we developed two recurrence prediction models at 5- and 10-year after surgery. The accuracy of prediction in test cases by each model were 100%. In addition, for predicting the time of recurrence during postoperative course, we combined the two models. The validation in test cases showed the accuracy of 100% in group A, B, and D patients. As shown in Fig. 2, there was a variability in tumor grade in test cases, which were predicted to have recurrence within 5 years (1st quadrant); nuclear grade in test cases were not always of high grade. The Fuhrman nuclear grading system has been the most used grading system for ccRCC. The grading is based on size, nucleolar prominence, and nuclear pleomorphism as microscopically observed by the pathologist. Recently, Um et al. reported that the replacement of nuclear grade with nuclear mean perimeter, measured by computational image analysis, can improve the accuracy of Leibovich score in the patients with localized ccRCC^10^. In the present study, we used 80 quantitative nuclear morphologic features obtained by digital images for developing recurrence prediction model. We believe that detailed digital information of nuclear morphologic feature can improve risk classification in the patients with localized RCC. In addition, the patients who had recurrence within 5 years showed various T stage and AUA risk grouping. Therefore, we believe that our novel recurrence prediction models are superior to T stage, Fuhrmann’s nuclear grade, and AUA risk grouping in predicting recurrence within 5 years after surgery.

On the contrary, inaccuracy of recurrence prediction after more than 5 years of surgery remains a priority issue for clinical management of postoperative surveillance. There was false prediction in 4 group C patients. In addition, 2 patients in group D had recurrence at 145 and 192 months after surgery (data not shown). Our recurrence prediction models use quantitative nuclear features that were tumor-related factors. This result suggests the limitation of recurrence prediction using only tumor-related factors.

We previously reported follow-up results for the postoperative neutrophil-to-lymphocyte ratio (NLR), which is an immune-related factor, and recurrence in patients with clear cell renal cell carcinoma^11^, demonstrating that the postoperative NLR was significantly decreased relative to the preoperative value, and that NLR at recurrence was significantly increased relative to the postoperative value. Therefore, we should include host-related factors such as sex, body mass index, immune-related factors, and the patient’s nutritional status in future prediction models, particularly those for predicting late recurrence^11–15^.

Although this system will be a useful tool for recurrence prediction for ccRCC, there are several limitations to generalize the results. First, the number of cases in the study is small for generalization. Additional cases will be required to improve accuracy of the models and prevent overfitting in machine learning. In addition, although we focused on only nuclear features of cancer cells in this study, other histological information, such as microvascular invasion, tumor necrosis, and lymphocytic infiltration as well as host-related factors. Regarding the selection of ROI, the part of tumor was selected manually in this study. To avoid selection bias, an automatic acquisition system might be necessary. However, we believe that the present study showed promising results that could contribute to the future development of artificial intelligence-based prediction model. The NCCN Guidelines version 2.2023 recommends adjuvant therapy using pembrolizumab for patients with stage 2 and 3 diseases. The precise prediction of recurrence within 5 years after surgery by our novel model would be useful for appropriate patients’ selection in clinical practice.

## Conclusion

This study demonstrated that SVM learning of nuclear morphological features in ccRCC can be used to create a new prediction model that is completely different from conventional models. The precise recurrence prediction within 5 years after surgery will improve postoperative management in the patients with ccRCC.

## Material and methods

### Patients

This retrospective study was conducted according to the ethical guidelines for clinical studies of the Ministry of Health, Labor and Welfare of Japan and approved by the Ethics Committee of Tokyo Medical University (approval number: T2019-0146). We had provided a public notice on our website regarding explanatory consent and the opportunity to refuse. Therefore, the need for informed consent was waived by the Ethics Committee of Tokyo Medical University.

We retrospectively reviewed the medical records of 349 patients with non-metastatic ccRCC (T1-3N0M0), who underwent radical or partial nephrectomy at our institution between 1990 and 2008. In order to develop 5-year and 10-year recurrence prediction model, one of the investigators of this study (A.S) selected a total of 131 patients based on the status of recurrence and follow-up period at the time of December 2013. The patients who were not followed-up for 5 years were excluded. Tumors were staged according to the 2002 Union Internationale Contre le Cancer TNM classification and graded according to the Fuhrman grading system^16, 17^. Pathological evaluation was performed by two senior pathologists (M.K. and T.N.). In principle all patients were followed-up by physical examination, blood evaluation and chest radiography at 3 months, and by computed tomography at 6 months. Other radiological studies were done as required. Our department also recommends follow-up as long as possible but does not mandate a follow-up of more than 10 years.

### Digital image processing for nuclear evaluation

**Figure 3:**
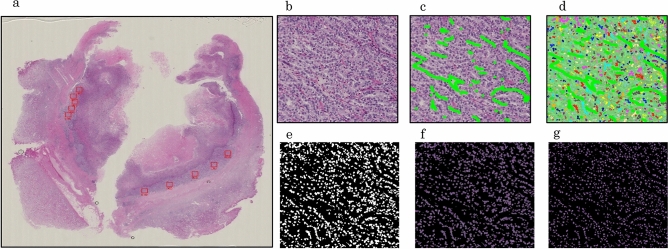
Image processing for extraction of nuclei. (**a**) Nine to 10 regions of interest (ROIs) are selected from one slide. (**b**) Each ROI is expanded. (**c**) Renal cell carcinomas (RCCs) other than clear cell RCCs are masked. (**d**) Nuclei are extracted. (**e**) A mask image with only the left nucleus is created. (**f**) The image in 3e is overlaid on the image in 3c. (**g**) Nucleus segmentation is performed using deep learning.

All hematoxylin and eosin (HE)-stained slides of ccRCC tissues were digitally recorded using a whole slide image Scanner (Nano Zoomer-RS: Hamamatsu Photonics, Hamamatsu, Japan) at × 20 image magnification. An average of 32 ROIs per case were selected by pathologist, excluding areas that were crushed, blurred, and areas infiltrated by numerous lymphocytes (Fig. 3a). A representative magnified image of ROI is shown in Fig. 3b. Each ROI contained fibroblasts and lymphocytes, and the area other than cancer areas were manually masked (Fig. 3c). We performed nuclei extraction for only RCC images (Fig. 3d) and created nuclei mask images (Fig. 3e) using Ilastik software (https://www.ilastik.org). By overlaying the image in Fig. 3e over the image in Fig. 3c, we obtained an image of RCC nuclei (Fig. 3f). At this stage, there were still many nuclei that were polymerized. The final image for nuclear measurement (Fig. 3g) was obtained by overlaying an additional nuclei segmentation mask created using pix2pix (https://phillipi.github.io/pix2pix/).

### Extraction of quantitative nuclear morphological information

Using CellProfiler software (https://cellprofiler.org), each nucleus was evaluated regarding nucleus shape related features (size, contour line length, roundness, maximum and minimum axis length, etc.) and chromatin texture features (entropy, second angular moment, variance, difference moment, etc.). The following CellProfiler Modules were employed: Measure Objects Size-Shape, Measure Texture, and Measure Object Radial Distribution. Details of the morphological features in CellProfiler can be found here: http://cellprofiler-manual.s3.amazonaws.com/CellProfiler-3.0.0/index.html. A graphical representation of the lateralized quantitative nuclear features is shown in Supplementary Fig. S1. CellProfiler outputted 80 features for each nucleus, and a total of 2,512,771 nuclei were measured. Finally, we employed the CFLCM method^18^, which shows the heterogeneity and pleomorphism of nuclei across an ROI image based on features of each nucleus. In this method, heterogeneity was calculated by treating the features of each cell nucleus as one pixel on the image. CFCLM used the data output by CellProfiler to output 960 features for each ROI, and a total of 4312 ROIs were measured.

### Development of recurrence prediction model using machine learning algorithm and the validation

**Figure 4:**
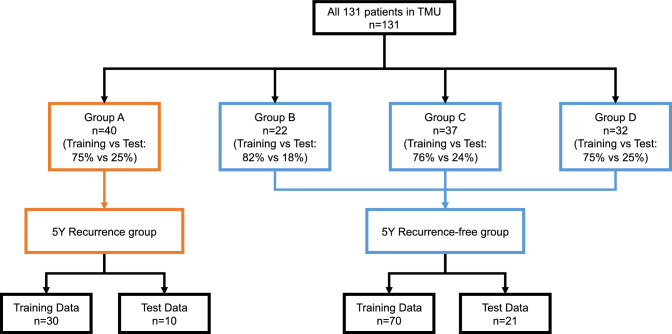
Inclusion of cases in the 5-year recurrence model.

We created two prediction model for recurrence within 5- and 10-years. As a machine learning method, we employed SVM. Data were analyzed using the statistical software package R version 3.6.1. We also used the package “e1071: SVM Linear Kernel”^19^. At first, we divided the data for 4312 ROIs (131 cases) into four groups according to recurrence and follow-up period: Group A, recurrence within 5 years; Group B, recurrence between 5 and 10 years; Group C, recurrence-free with 5–10 years of follow-up; and Group D, recurrence-free for more than 10 years of follow-up. The number of cases in each group was 40, 22, 37, and 32, respectively. Test data were randomly selected from each group. Data for a total of 31 cases, including 10, four, nine, and eight from Groups A, B, C, and D, respectively, were separated as test data, and the rest were used as SVM model training data. In the 5-year recurrence model, Group A data were recurrence data whereas Group B, C, and D data were recurrence-free data. The number of cases were as follows: recurrence, 40 (training 30 and test 10); recurrence-free, 91 (training 70 and test 21; Fig. 4).

**Figure 5:**
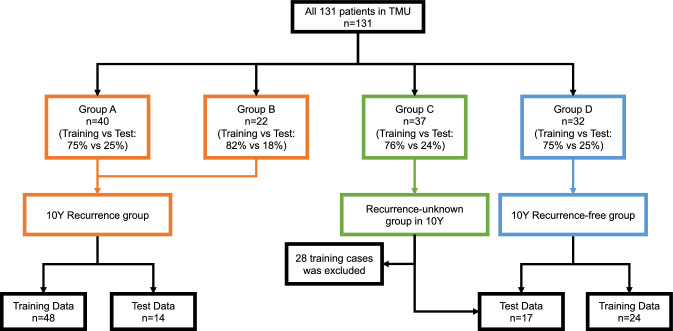
Inclusion of cases in the 10-year recurrence model.

With regard to the 10-year model, Groups A and B were the recurrence groups while Group D was the recurrence-free group. Data for all cases in Group C could not be used as training data, although four cases from Group C that were included as test cases in the 5-year model were included as test cases in the 10-year model, which then included 14 test cases (Fig. 5). In both models, the total cases were randomly divided into training and test sets (3:1). We used the average of each ROI recurrence probability, which were outputted by SVM, as the result of prediction models. The accuracy of the models was confirmed by validation for test cases with each model. Finally, to evaluate the time of recurrence in the postoperative course (recurrence within 5 years, recurrence between 5 and 10 years, and recurrence-free within 10 years after surgery), we created a plot of test cases according to the calculated recurrence probabilities by two models. We also checked the distribution of T stage, nuclear grade, and AUA risk group for follow-up on the plot. Furthermore, we validated the accuracy of prediction in the test cases using follow-up data from December 2021.

## Supplementary Information

Supplementary Tables.

Supplementary Figure S1.

## Data Availability

Data are available from the corresponding author upon reasonable request.
